# Identification of key biomarkers associated with cell adhesion in multiple myeloma by integrated bioinformatics analysis

**DOI:** 10.1186/s12935-020-01355-z

**Published:** 2020-06-22

**Authors:** Yue Peng, Dong Wu, Fangmei Li, Peihua Zhang, Yuandong Feng, Aili He

**Affiliations:** Department of Hematology, The Second Affiliated Hospital of Xi’an Jiaotong University, 157, 5th West Road, 710004 Xi’an, Shaanxi China

**Keywords:** Bioinformatics analysis, Multiple myeloma, Biomarker

## Abstract

**Background:**

Multiple Myeloma (MM) is a hematologic malignant disease whose underlying molecular mechanism has not yet fully understood. Generally, cell adhesion plays an important role in MM progression. In our work, we intended to identify key genes involved in cell adhesion in MM.

**Methods:**

First, we identified differentially expressed genes (DEGs) from the mRNA expression profiles of GSE6477 dataset using GEO2R with cut-off criterion of p < 0.05 and [logFC] ≥ 1. Then, GO and KEGG analysis were performed to explore the main function of DEGs. Moreover, we screened hub genes from the protein–protein interaction (PPI) network analysis and evaluated their prognostic and diagnostic values by the PrognoScan database and ROC curves. Additionally, a comprehensive analysis including clinical correlation analysis, GSEA and transcription factor (TF) prediction, pan-cancer analysis of candidate genes was performed using both clinical data and mRNA expression data.

**Results:**

First of all, 1383 DEGs were identified. Functional and pathway enrichment analysis suggested that many DEGs were enriched in cell adhesion. 180 overlapped genes were screened out between the DEGs and genes in GO terms of cell adhesion. Furthermore, 12 genes were identified as hub genes based on a PPI network analysis. ROC curve analysis demonstrated that ITGAM, ITGB2, ITGA5, ITGB5, CDH1, IL4, ITGA9, and LAMB1 were valuable biomarkers for the diagnosis of MM. Further study demonstrated that ITGA9 and LAMB1 revealed prognostic values and clinical correlation in MM patients. GSEA and transcription factor (TF) prediction suggested that MYC may bind to ITGA9 and repress its expression and HIF-1 may bind to LAMB1 to promote its expression in MM. Additionally, pan-cancer analysis showed abnormal expression and clinical outcome associations of LAMB1 and ITGA9 in multiple cancers.

**Conclusion:**

In conclusion, ITGA9 and LAMB1 were identified as potent biomarkers associated with cell adhesion in MM.

## Background

MM is known as a plasma cell malignancy with an unlimited proliferation of abnormal plasma cells in the BM and high levels of monoclonal protein in the blood and urine [1]. The advance of novel chemotherapeutic agents and cell therapy brings great improvements for MM patients. However, it remains largely incurable because of relapse and resistance [2, 3]. Therefore, searching for novel prognostic biomarkers and therapeutic targets is crucial for MM patients.

Previous studies have demonstrated the close interaction between myeloma cells and the BM microenvironment. The BM microenvironment included ECM components that composed of fibronectin, laminin, collagen and a variety of cellular residents that consisted of bone marrow stromal cells, hematopoietic stem cells, immune cells, etc. [4]. MM cells and the BM microenvironment interacted through a complex network mediated by cytokines and adhesion molecules, including the integrins, the immunoglobulin superfamily, the selectins, the cadherins, and the proteoglycans. The cross-linking activated several known regulatory pathways, which involved in the survival, proliferation, migration, homing, as well as drug resistance of MM cells, playing a crucial role in MM development [5].

Integrins are heterodimeric membrane glycoproteins expressing on the surface of many types of cells, serving as CAMs and the major receptors for the ECM [5, 6]. Their expression can vary considerably between normal and cancer tissues. Studies showed that integrin expression levels were correlated with pathological outcomes, including patient survival and metastasis in many cancer types. Furthermore, Integrins regulated a series of cellular functions such as cell proliferation, migration, invasion, and survival which play a crucial role in the cancer progression [7, 8]. ITGA9 is one of the least studied integrins among the 24-member integrin family in human. Furthermore, studies have suggested that ITGA9 had the abnormal expression in numerous cancers and has been found to be crucial for a number of biological processes in many types of cancers, such as breast carcinoma, melanoma and lung cancer [9–12].

Laminins are extracellular heterotrimeric basement membrane glycoproteins [13]. They are composed of three polypeptide chains, named as α, β, and γ [14]. Laminins are involved in diverse physiological and pathological processes, including involvement in basement membrane assembly, neurite outgrowth, and promotion of cell adhesion, migration, protease activity, proliferation and angiogenesis in cancers [15, 16]. The link between cancer cells and laminins is vital in tumor invasion and metastasis. Invading tumor cells interact with laminins to acquire more metastatic potential [17]. LAMB1 is expressed in most tissues and is one of the 3 chains constituting laminin 1. Studies have shown that LAMB1 was shown to be a potential biomarker in some cancers [18, 19].

However, there is rare research on the role which ITGA9 and LAMB1 play in myeloma development and metastasis. With the development of gene expression profiling array and second-generation high-throughput sequencing technology, people analyze the data on gene expression profiles to screen DEGs to find candidate biomarkers and potential therapeutic targets in MM [20, 21].In this study, we aim to find potential biomarkers in MM. First, we analyzed a microarray profile to obtain DEGs between active MM and normal plasma cells. Then, 12 genes were identified as hub genes based on a PPI network analysis. Finally, through integrative bioinformatic analysis, we identified that ITGA9 and LAMB1 were correlated with cell adhesion in MM.

## Materials and methods

### Microarray data information

Three gene expression profiles (GSE6477, GSE2658 and GSE136324) were from the GEO database (http://www.ncbi.nlm.nih.gov/geo). The GSE6477 dataset, which included samples from 147 patients with different stages of plasma cell neoplasm and 15 NDs, was performed on the Affymetrix Human Genome U133A Array platform. The GSE2658 dataset was composed of 559 samples of MM patients. 426 patients with MM was obtained from the GSE136324 dataset. Both of them were performed on the Affymetrix Human Genome U133 Plus 2.0 Array. All the above-mentioned profiling datasets were accessible online with no conflict.

### Identification of DEGs in MM

The online analysis tool GEO2R was used to screen DEGs of the GSE6477 dataset. In this study, we set “p < 0.05 and [logFC] ≥ 1” as the cut-off criterion. A heatmap of the top 100 significantly changed DEGs were drawn using the Multiple Experiment Viewer software.

### GO and KEGG enrichment analysis

GO enrichment analysis (http://www.geneontology.org/) and KEGG pathway enrichment analysis (http://www.genome.jp/kegg/pathway.html) were used to illuminate the biological functions and pathways associated with DEGs [16], which were both integrated in the DAVID (http://david.abcc.ncifcrf.gov/) program. Enriched GO and KEGG terms with p < 0.05 were considered as statistical significance. The ClueGO and CluePedia plug‐in Cytoscape software version 3.7.1 (http://www.cytoscape.org/) were used to analyze the pathways interrelation.

### PPI network construction

STRING (http://string-db.org) online database is used to predict the PPI information. The cut-off value for the filtration criteria was set at the confidence score > 0.7. Then, the PPI network was visualized by Cytoscape. The MCODE plugin in Cytoscape was applied to select modules of the PPI network.

### ROC analysis

The ROC curves were used to explore the sensitivity and specificity of DEGs for MM diagnosis using GraphPad Prism Software (Version V, La Jolla, CA, USA).

### Survival analysis

The PrognoScan database (http://dna00.bio.kyutech.ac.jp/PrognoScan/) was used to investigate the prognostic impact of selected genes in different cancers. According to the median expression of a particular gene, the patients were split into high and low expression groups. The OS and DSS of GC patients was evaluated using a KM plot. A Cox *p* value<0.05 was considered as statistically significant.

### Analysis of expression in various cancers

The mRNA expression levels of LAMB1 and ITGA9 in various cancers and their normal tissue counterparts were analyzed using the Oncomine database (https://www.oncomine.org/resource/login.html). The relationship between two specific gene mRNA expression levels in various cancers were analyzed using the GEPIA (Gene Expression Profiling Interactive Analysis) database (http://gepia.cancer-pku.cn/).

### GSEA

GSEA version 2.2.1 software was used to analyze genes function from the GSEA website MSIGDB database (http://software.broadinstitute.org/gsea/msigdb). The default weighted enrichment method was applied for enrichment analysis. The random combination was set for 1000 times. The analysis was performed with following settings: FDR<0.25, NOM p-value<0.05 and |NES|>1. The enrichment map plugin in Cytoscape was applied to visualize geneset enrichment results.

### Statistical analysis

An independent sample t-test and one-way ANOVA were used to compare the statistical significance between two or more samples, respectively. Two-tailed p-values < 0.05 were considered as statistically significance. The correlations analysis was assessed by the Spearman correlation coefficient and Chi square test. Statistical analysis was performed by the SPSS 22.0 and GraphPad Prism Software.

## Results

### Identification of DEGs

**Fig. 1:**
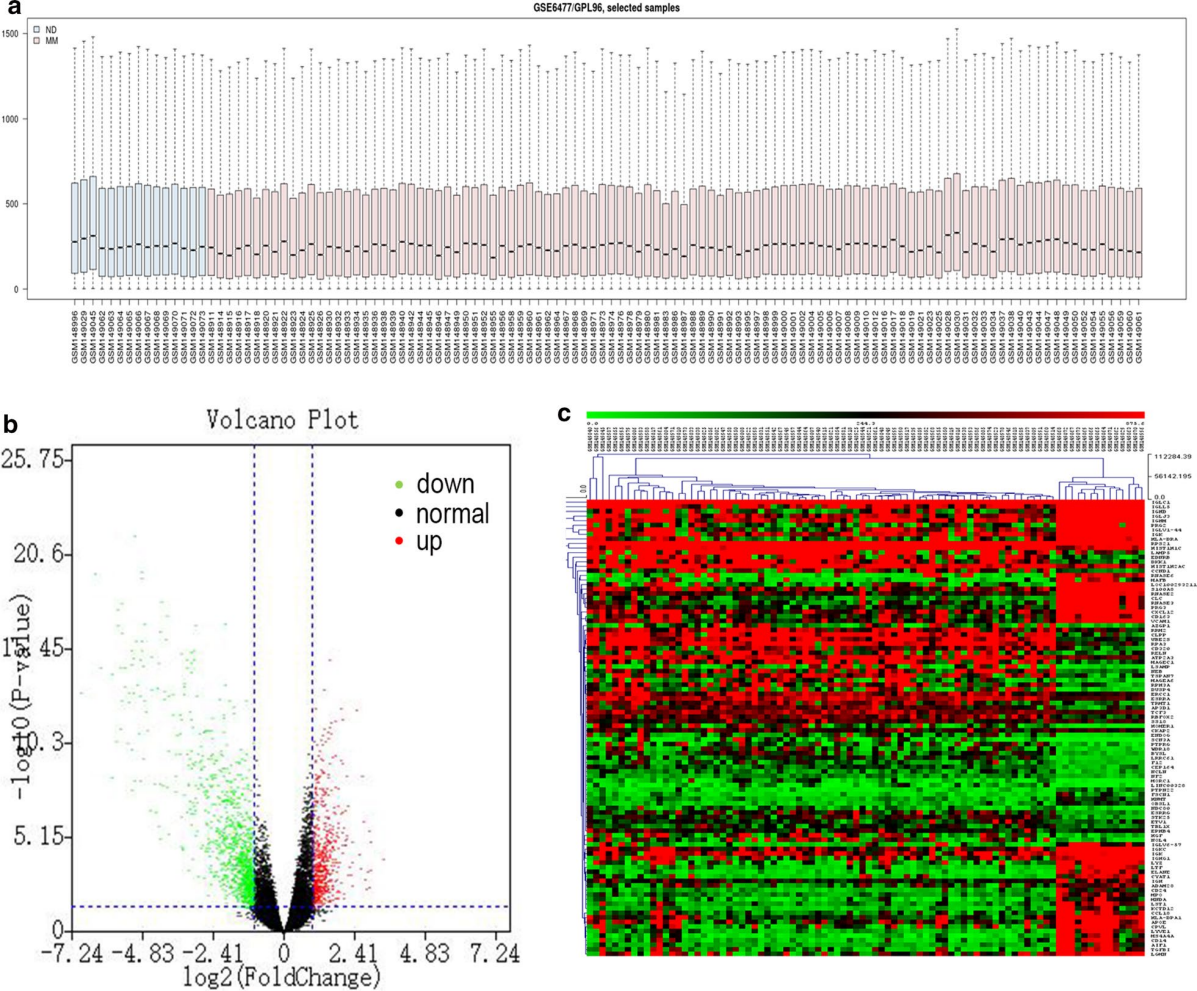
Identification of DEGs. **a** Boxplot analysis of new and relapsed MM patients and normal donors in the GSE6477 datasets. **b** Respective volcano plot of the GSE6477 datasets. Red and green plots represent genes with [logFC] ≥ 1 and p < 0.05. Black plots represent the remaining genes with no significant difference. **c** Heatmap of the top 100 DEGs (50 up-regulated and 50 down-regulated genes)

We acquired mRNA expression profiles of plasma cell samples from 101 patients of new or relapse multiple myeloma and 15 NDs from the GSE6477. Gene expression distribution of profiles was matched via boxplot analysis (Fig. 1a). Statistical analysis was required to validate the comparison between NDs and MM patients. We applied the GEO2R to identify DEGs associated with MM. 1383 DEGs (538 upregulated genes and 845 downregulated genes) were identified in the MM samples compared to NDs’ samples. We set the cut‐off criterion as p < 0.05 and [logFC] ≥ 1 (Fig. 1b). The top 100 genes were clustered in the heatmap between patients with MM patients and NDs (Fig. 1c).

### Functional and pathway enrichment analysis

**Fig. 2:**
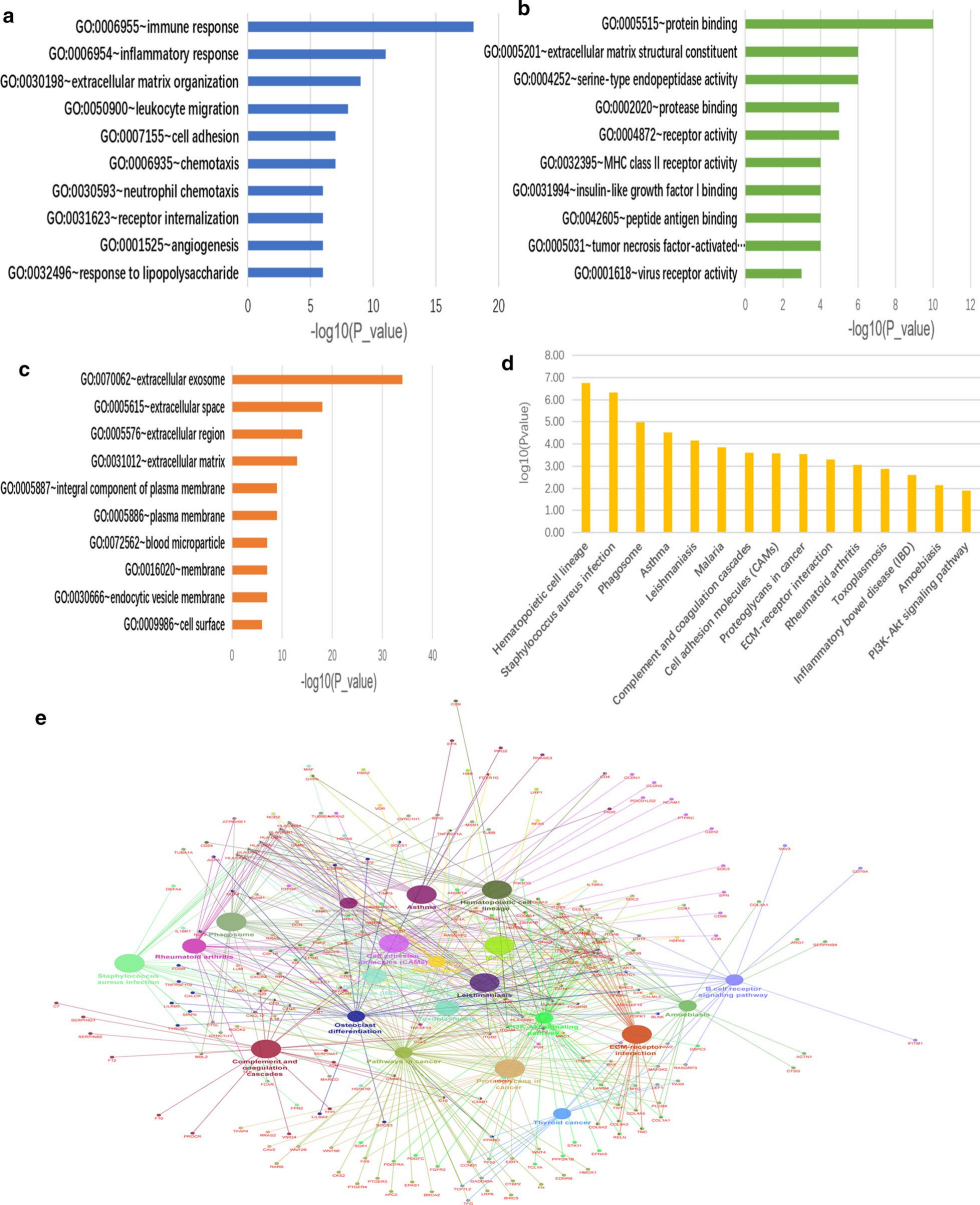
Functional and pathway enrichment analysis. (**a–c**) The GO enrichment analysis of DEGs, including BP (**a**), MF (**b**) and CC (**c**). **d** The KEGG enrichment analysis of DEGs. **e** Interrelation analysis of pathways and related genes via assessment of KEGG processes in ClueGO and CluePedia

We performed GO and KEGG enrichment analysis to investigate the functions of DEGs using DAVID. The top GO (Fig. 2a–c, Table 1) and KEGG (Fig. 2d, Table 2) terms for DEGs were shown. For biological process (BP), DEGs were mainly enriched in immune response, inflammatory response, ECM organization, leukocyte migration, cell adhesion (Fig. 2a). DEGs in molecular function (MF) were significantly associated with protein binding, ECM structural constituent, serine type endopeptidase activity, protease binding and receptor activity (Fig. 2b). The cellular components (CC) analysis indicated that proteins encoded by DEGs were mostly located in the extracellular exosome, extracellular space, extracellular region and ECM (Fig. 2c). KEGG enrichment analysis showed that CAMs, proteoglycans in cancer, ECM-receptor interaction and PI3K/Akt signaling pathway were significantly enriched in DEGs. The top KEGG pathways for DEGs were shown (Fig. 2d). Subsequently, we analyzed a pathway interrelation and related genes by examining KEGG enrichment analysis results in ClueGO and CluePedia (Fig. 2e). These results suggested that cell adhesion might play a crucial part in the malignant progression of MM.

**Table 1:** Top 30 enriched GO terms of DEGs

Category	Term	Count	Percentage (%)	P-value
GOTERM_CC_DIRECT	GO:0070062 ~ extracellular exosome	360	18.29966	1.26E − 34
GOTERM_CC_DIRECT	GO:0005615 ~ extracellular space	182	9.251493	4.94E − 19
GOTERM_BP_DIRECT	GO:0006955 ~ immune response	87	4.422417	7.17E − 19
GOTERM_CC_DIRECT	GO:0005576 ~ extracellular region	195	9.912314	4.68E − 15
GOTERM_CC_DIRECT	GO:0031012 ~ extracellular matrix	60	3.049943	1.21E − 13
GOTERM_BP_DIRECT	GO:0006954 ~ inflammatory response	68	3.456602	9.07E − 12
GOTERM_MF_DIRECT	GO:0005515 ~ protein binding	733	37.26013	8.97E − 11
GOTERM_BP_DIRECT	GO:0030198 ~ extracellular matrix organization	42	2.13496	6.27E − 10
GOTERM_CC_DIRECT	GO:0005887 ~ integral component of plasma membrane	159	8.082349	8.46E − 10
GOTERM_CC_DIRECT	GO:0005886 ~ plasma membrane	375	19.06214	1.60E − 09
GOTERM_BP_DIRECT	GO:0050900 ~ leukocyte migration	30	1.524971	9.93E − 09
GOTERM_CC_DIRECT	GO:0072562 ~ blood microparticle	32	1.626636	4.62E − 08
GOTERM_CC_DIRECT	GO:0016020 ~ membrane	216	10.97979	6.05E − 08
GOTERM_CC_DIRECT	GO:0030666 ~ endocytic vesicle membrane	20	1.016648	6.35E − 08
GOTERM_BP_DIRECT	GO:0007155 ~ cell adhesion	67	3.40577	7.87E − 08
GOTERM_BP_DIRECT	GO:0006935 ~ chemotaxis	28	1.423307	1.55E − 07
GOTERM_CC_DIRECT	GO:0009986 ~ cell surface	71	3.609099	3.34E − 07
GOTERM_CC_DIRECT	GO:0009897 ~ external side of plasma membrane	37	1.880798	5.70E − 07
GOTERM_MF_DIRECT	GO:0005201 ~ extracellular matrix structural constituent	19	0.965815	6.40E − 07
GOTERM_BP_DIRECT	GO:0030593 ~ neutrophil chemotaxis	19	0.965815	7.14E − 07
GOTERM_BP_DIRECT	GO:0031623 ~ receptor internalization	15	0.762486	1.18E − 06
GOTERM_MF_DIRECT	GO:0004252 ~ serine-type endopeptidase activity	41	2.084128	1.88E − 06
GOTERM_BP_DIRECT	GO:0001525 ~ angiogenesis	38	1.93163	2.05E − 06
GOTERM_CC_DIRECT	GO:0005925 ~ focal adhesion	54	2.744949	2.13E − 06
GOTERM_CC_DIRECT	GO:0005829 ~ cytosol	294	14.94472	2.33E − 06
GOTERM_BP_DIRECT	GO:0032496 ~ response to lipopolysaccharide	31	1.575804	2.46E − 06
GOTERM_BP_DIRECT	GO:0060326 ~ cell chemotaxis	18	0.914983	2.73E − 06
GOTERM_BP_DIRECT	GO:0050729 ~ positive regulation of inflammatory response	19	0.965815	3.52E − 06
GOTERM_CC_DIRECT	GO:0005578 ~ proteinaceous extracellular matrix	41	2.084128	3.62E − 06

**Table 2:** Top 15 enriched KEGG terms of DEGs

Term	P-value	Percentage (%)	Count
KEGG:04640 Hematopoietic cell lineage	0.00000	30.93	30
KEGG:05150 *Staphylococcus aureus* infection	0.00000	35.29	24
KEGG:04145 Phagosome	0.00000	23.68	36
KEGG:05310 Asthma	0.00000	45.16	14
KEGG:05140 Leishmaniasis	0.00000	29.73	22
KEGG:05144 Malaria	0.00000	34.69	17
KEGG:04610 Complement and coagulation cascades	0.00000	27.85	22
KEGG:04514 Cell adhesion molecules (CAMs)	0.00000	22.22	32
KEGG:05205 Proteoglycans in cancer	0.00000	19.90	40
KEGG:04512 ECM-receptor interaction	0.00000	26.83	22
KEGG:05323 Rheumatoid arthritis	0.00000	25.27	23
KEGG:05145 Toxoplasmosis	0.00001	23.01	26
KEGG:05321 Inflammatory bowel disease (IBD)	0.00001	27.69	18
KEGG:05146 Amoebiasis	0.00003	22.92	22
KEGG:04151 PI3K-Akt signaling pathway	0.00006	15.54	55

### PPI network and modular analysis of selected genes

**Fig. 3:**
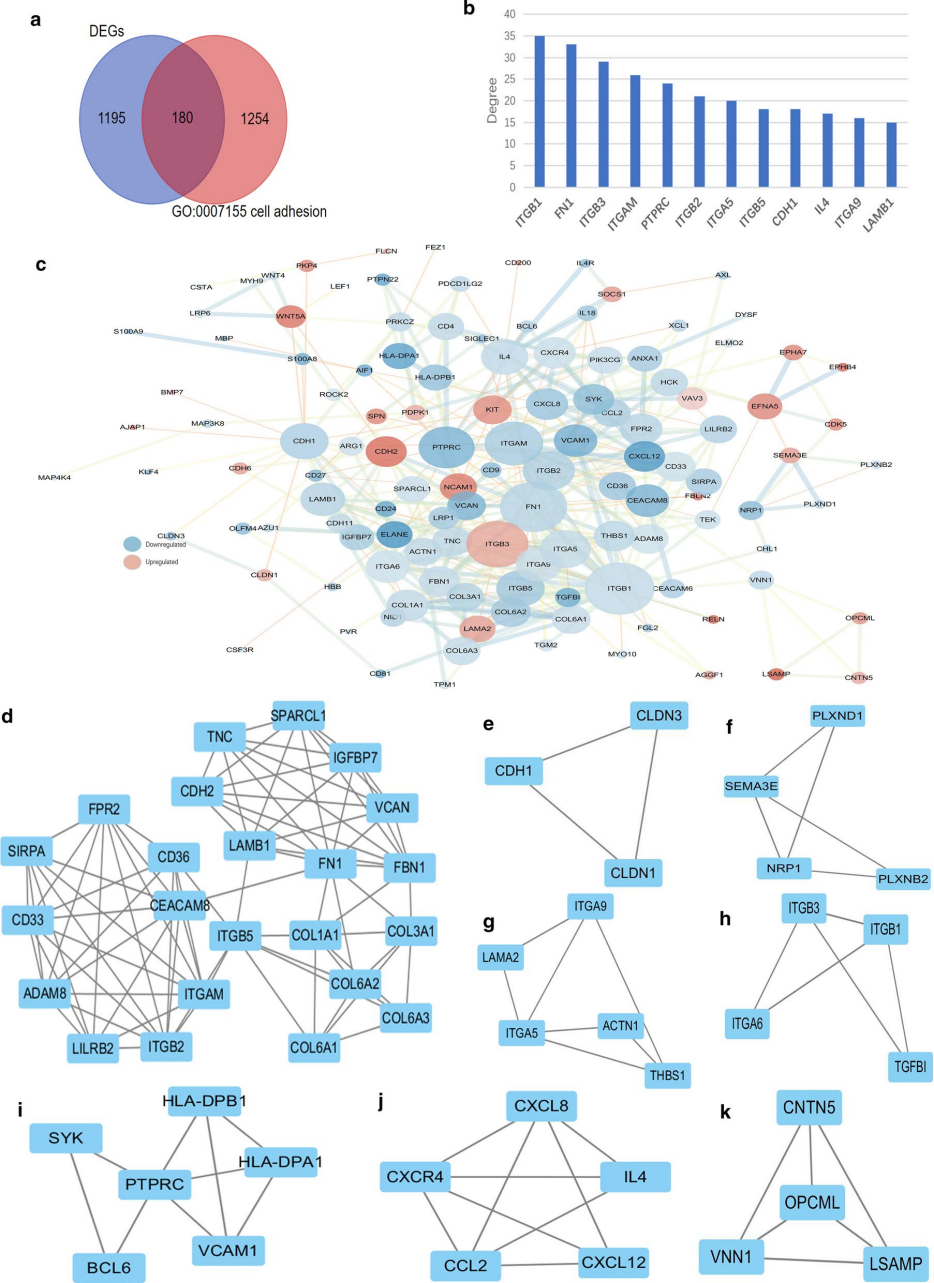
PPI network and modular analysis of selected genes. **a** The venn graph of DEGs and genes in GO term of cell adhesion. **b** The degree value of the top 12 hub genes. **c** PPI network constructed by 123 selected proteins which are associated with cell adhesion with minimum required interaction score at 0.7. The diameter and color of the nodes represented the value of the degree and logFC respectively. The thickness and color of the edges represented the combined scores. (**d–k**) Eight modules generated by the MCODE

To identify key genes in the cell adhesion processes, 180 overlapped genes in DEGs and genes involved with the cell adhesion were screened, including 123 mRNAs and 57 non-coding RNAs (Fig. 3a). Then, we constructed a PPI network containing 123 protein, with 123 nodes and 429 edges based on the STRING database and Cytoscape (Fig. 3c). 12 hub genes whose degree values ≥ 15 for further analysis were chosen, including ITGB1, FN1, ITGB3, ITGAM, PTPRC, ITGB2, ITGA5, ITGB5, CDH1, IL4, ITGA9 and LAMB1 (Fig. 3b). Additionally, Cytoscape displayed a total of eight modules in the default MCODE settings for modular analysis (Fig. 3d–k, Table 3).

**Table 3:** Modules obtained from the PPI network

Cluster	Score (Density*#Nodes)	Nodes	Edges	Node IDs
1	7.818	23	86	ADAM8, FPR2, VCAN, COL6A1, COL6A2, IGFBP7, CD33, CDH2, SPARCL1, FN1, LILRB2, COL1A1, COL3A1, ITGAM, ITGB2, CD36, TNC, CEACAM8, ITGB5, COL6A3, LAMB1, FBN1, SIRPA
2	4.5	5	9	CXCL8, CXCR4, CCL2, CXCL12, IL4
3	4	4	6	LSAMP, VNN1, OPCML, CNTN5
4	3.6	6	9	BCL6, HLA-DPB1, HLA-DPA1, VCAM1, PTPRC, SYK
5	3.5	5	7	ITGA5, THBS1, LAMA2, ACTN1, ITGA9
6	3.333	4	5	TGFBI, ITGA6, ITGB1, ITGB3
7	3.333	4	5	PLXNB2, NRP1, PLXND1, SEMA3E
8	3	3	3	CLDN3, CLDN1, CDH1

### Diagnostic and prognostic values of hub genes in MM

**Fig. 4:**
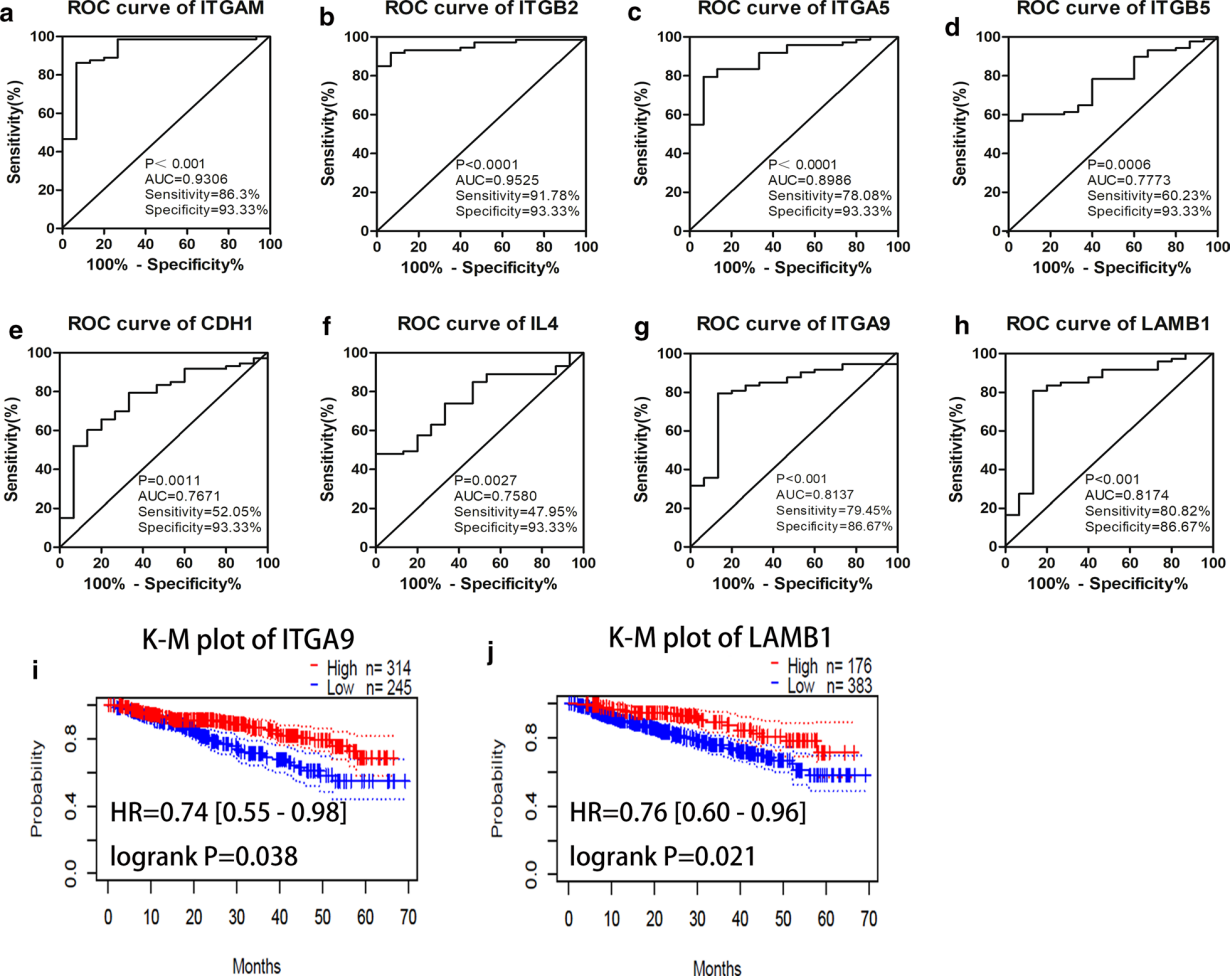
Diagnostic and prognostic values of hub genes in MM. **a–h** The ROC curves of ITGAM, ITGB2, ITGA5, ITGB5, CDH1, IL4, ITGA9, and LAMB1 in diagnosing MM of GSE2658 dataset. **i–j** The K-M survival curves comparing high and low expression of ITGA9 (**i**) and LAMB1 (**j**) in MM based on the GSE2658 dataset

First of all, we performed the ROC curve analysis among 12 hub genes based on the GSE2658. The results showed that ITGAM, ITGB2, ITGA5, ITGB5, CDH1, IL4, ITGA9, and LAMB1 achieved an AUC value of > 0.7, demonstrating that these eight genes have high sensitivity and specificity for MM, suggesting they can be served as biomarkers for the diagnosis of MM (Fig. 4a–h).

Furthermore, we evaluated the influence of all aforementioned 8 hub genes on clinical prognosis using PrognoScan database based on the GSE2658 through K-M curve and log-rank test to identify whether these genes were concerned with the survival of MM patients. ITGA9 (Fig. 4i) and LAMB1 (Fig. 4j) were significantly associated with DSS in MM by evaluating the correlation between these gene expressions and survival rates.

### The relationship between ITGA9/LAMB1 mRNA expression and clinical characters of patients with MM

**Fig. 5:**
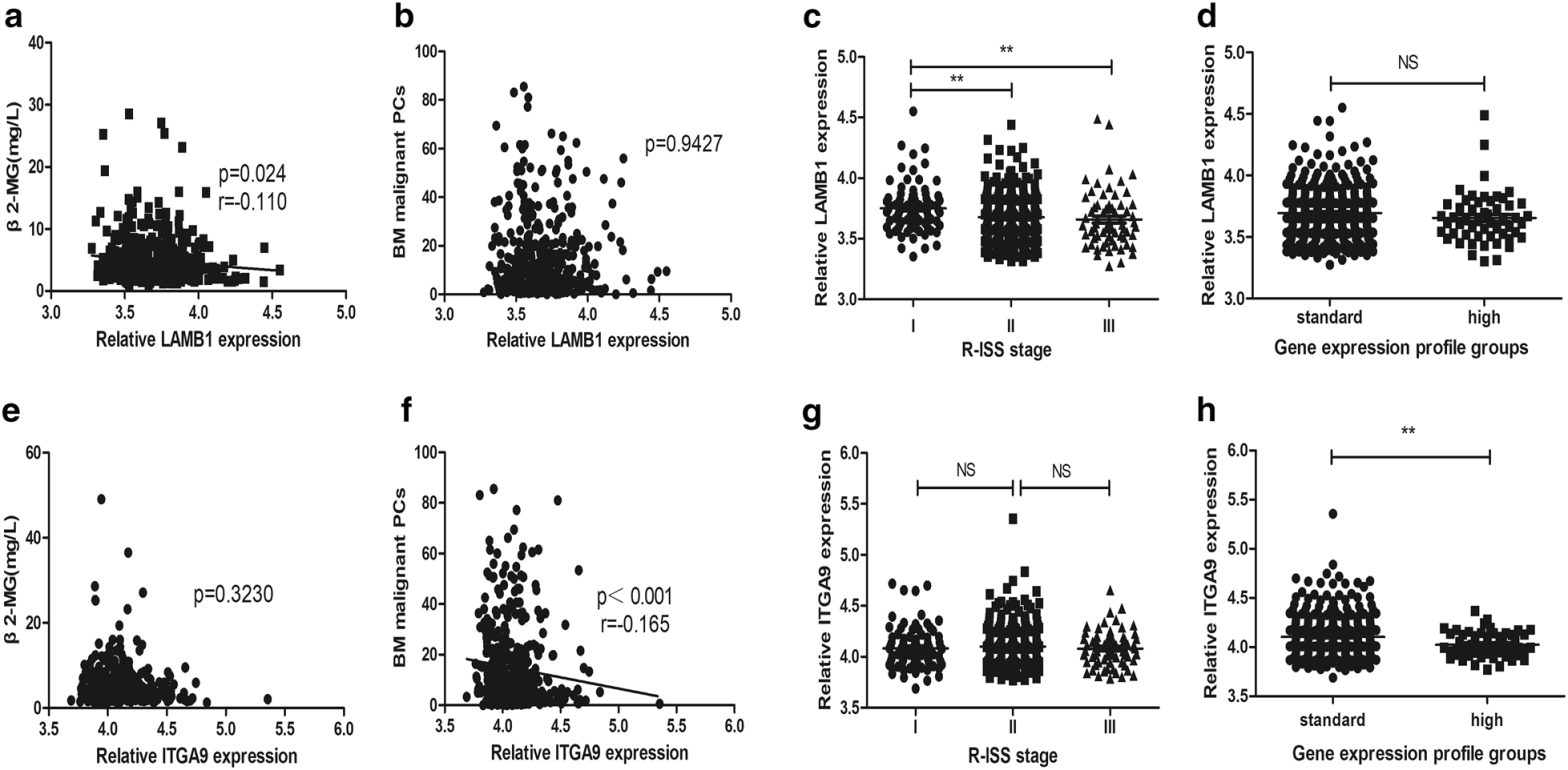
The relationship between ITGA9/LAMB1 mRNA expression and clinical characters of patients with MM. **a** LAMB1 mRNA expression had a negative correlation with β2-MG. **b** No significant relationship was found between LAMB1 mRNA expression and the count of malignant plasma cells in BM. **c** LAMB1 mRNA expression in the R-ISS II or III stage was much lower compared with the expression in R-ISS I stage. **d**There was no significant difference in LAMB1 mRNA expression between GEP high and standard group. **e** There is no significant relationship between ITGA9 mRNA expression and β2-MG. **f** ITGA9 mRNA expression had a negative correlation with the count of malignant plasma cells in BM. **g** ITGA9 mRNA expression was not correlated to the R-ISS stage. **h** LAMB1 mRNA expression in the GEP high group was much lower than that in the GEP standard group

First of all, the relationship between ITGA9 or LAMB1 mRNA expression and the clinical parameters was analyzed based on both clinical data and mRNA expression data of GSE136324 (Table 4, n = 426). The results showed that LAMB1 expression was negatively correlated with β_2_-MG (Fig. 5a, p = 0.024, spearman correlation coefficient r = -0.110). Patients with low LAMB1 expression had a tendency of being in high R-ISS stage (Fig. 5c, p = 0.001). No significant relationship was found between LAMB1 mRNA expression and malignant plasma cells (PCs) in BM (Fig. 5b, p = 0.9427) and GEP groups (Fig. 5d, p = 0.1785).

**Table 4:** The relationship between ITGA9/LAMB1 expression and clinical parameters of patients with MM

Character	Low ITGA9 group	High ITGA9 group	P-value	Low LAMB1 group	High LAMB1 group	P-value
Gender (M/F)	120/86	137/74	0.097	125/83	132/77	0.294
Age	57.90 ± 0.66	59.37 ± 0.57	0.173	58.57 ± 0.63	58.71 ± 0.61	0.913
ISS			0.641			0.219
I	87	81		77	91	
II	63	72		67	68	
III	62	59		68	53	
R-ISS			0.896			**0.001**
I	43	40		30	53	
II	132	138		141	129	
III	33	33		38	28	
LDH (mean, IU/l) (± SD)	169.46 ± 6.68	156.59 ± 3.24	0.628	164.98 ± 5.07	160.92 ± 5.38	0.290
β_2_m (mean, mg/L) (± SD)	5.220.35	4.92 ± 0.29	0.966	5.36 ± 0.34	4.78 ± 0.30	**0.024**
BM malignant PCs (mean, %) (± SD)	16.53 ± 1.15	13.85 ± 1.18	**0.001**	15.89 ± 1.26	14.48 ± 1.06	0.824
GEP (Standard/High)	180/33	193/20	**0.039**	182/31	191/22	0.120

*LDH* lactate dehydrogenase, *β2-MG* β2-microglobulin, *SD* standard deviation

Bold values indicate the statistical significant P-value

Furthermore, there was a negative correlation between ITGA9 expression and malignant PCs in BM (Fig. 5f, p < 0.001, spearman correlation coefficient r = −0.165). Compared with in the standard group, MM patients in the GEP high group had a lower ITGA9 expression (Fig. 5h, p = 0.0039). ITGA9 mRNA expression had no correlation with β-2MG (Fig. 5e, p = 0.323) and R-ISS stage (Fig. 5g, p = 0.281).

### Function and signaling pathways analysis of ITGA9 and LAMB1

To investigate the function of ITGA9 and LAMB1 on MM progression, we performed a comprehensive analysis including GSEA, TF prediction and Spearman correlation analysis.

**Fig. 6:**
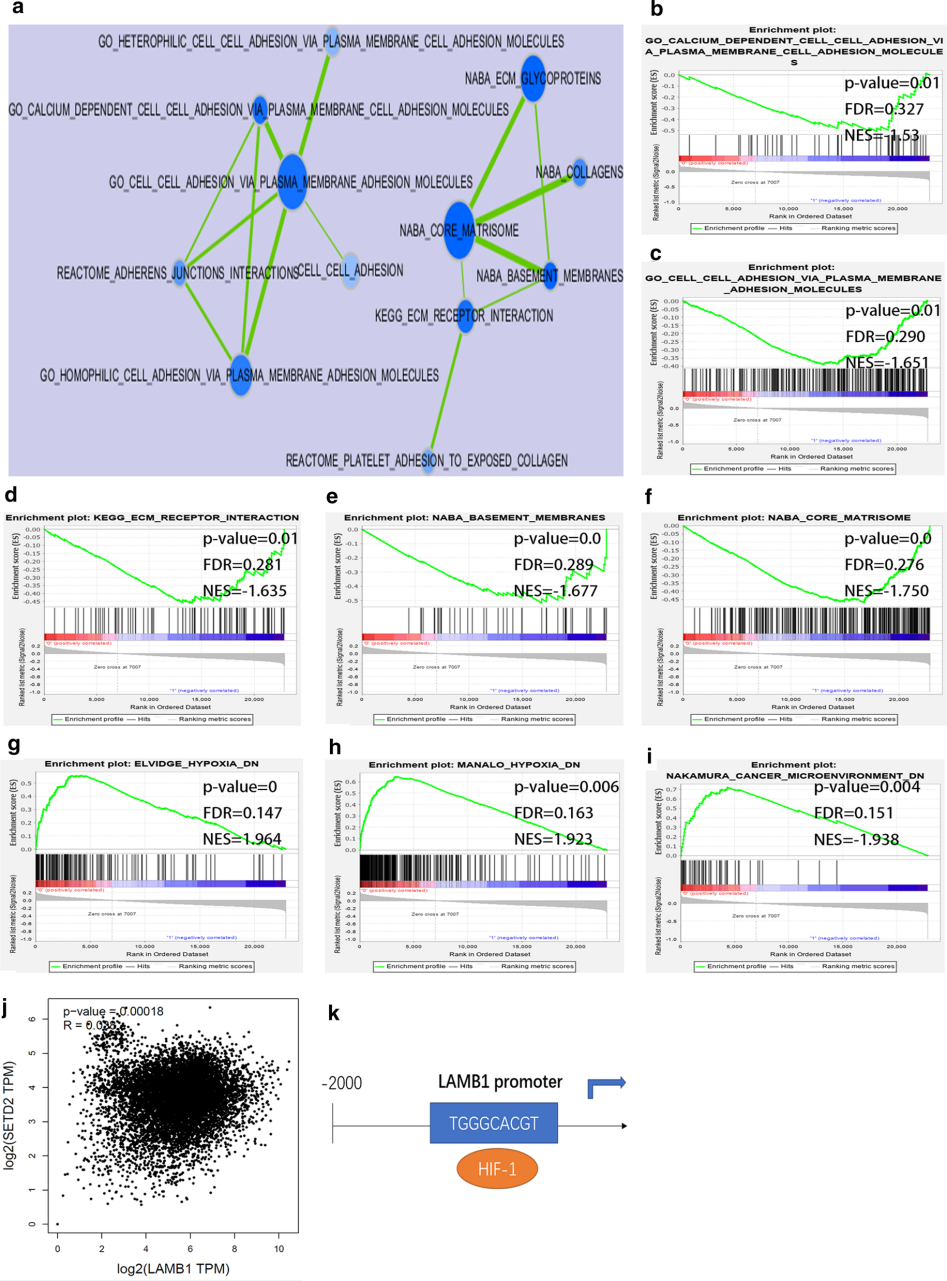
Function and signaling pathways analysis of LAMB1. **a** An enrichment map was constructed using gene sets with p‐value < 0.05. **b–i** The GSEA analysis between the low and high levels of LAMB1 in MM using the gene set in GSE2658 datasets. **j** The relationship between mRNA expression of LAMB1 and HIF-1. **k** The schematic representation of the position where the LAMB1 promoter may bind to HIF-1

Firstly, an enrichment map was constructed using genesets which related to cell adhesion with p‐value < 0.05 (Fig. 6a). Genesets including GO calcium dependent cell cell adhesion via plasma membrane cell adhesion molecules, GO cell adhesion via plasma membrane adhesion molecules, KEGG ECM receptor interaction, NABA basement membranes, NABA core matrisome were enriched in patients with low LAMB1 expression in the GSE2658, suggesting that LAMB1 was involved in the cell adhesion in myeloma cells (Fig. 6b–f). Furthermore, cancer microenvironment- dn, MANALO hypoxia dn, ELVIDGE hypoxia dn genesets were significantly enriched in patients with low LAMB1 expression in the GSE2658 (Fig. 6g–i). For futher study, we found the positive correlation between LAMB1 and HIF-1 mRNA expression in a varity of cancers using GEPIA database (Fig. 6j, p = 0.00018, spearman correlation coefficient r = 0.038). The result of TF prediction by the online site PROMO (http://alggen.lsi.upc.es/cgi-bin/promo_v3/promo/promoinit.cgi?dirDB=TF_8.3/) strengthened the correlation that HIF-1 may bind to the promoter of LAMB1 (Fig. 6k). Therefore, LAMB1 which may be regulated by HIF-1 played a vital role in myeloma cell adhesion.

**Fig. 7:**
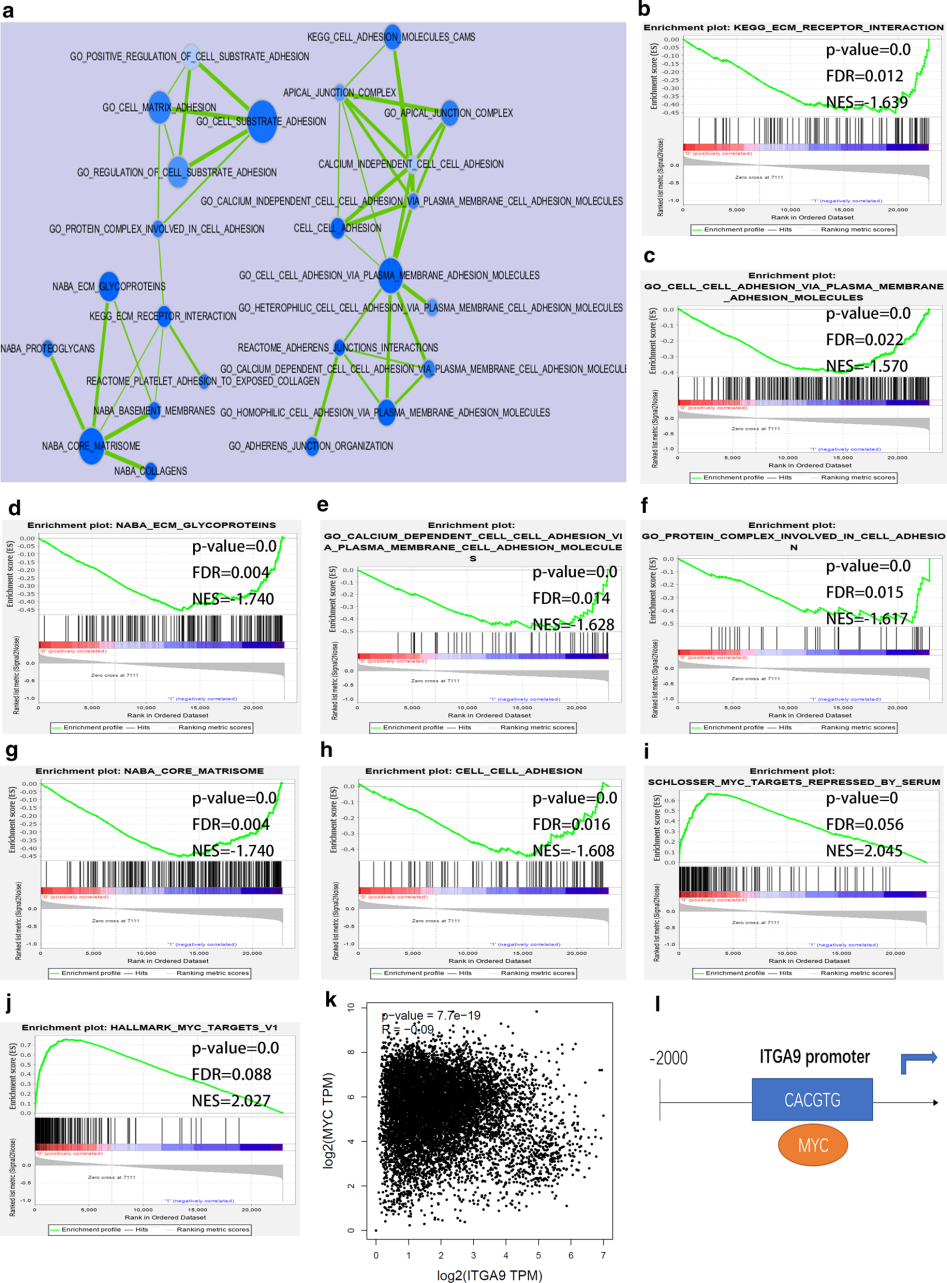
Function and signaling pathways analysis of ITGA9. **a** An enrichment map was constructed using gene sets with p‐value < 0.05. **b–j** The GSEA analysis between the low and high levels of ITGA9 in MM using the gene set in GSE2658 datasets. **k** The relationship between mRNA expression of ITGA9 and MYC. **l** The schematic representation of the position where the ITGA9 promoter may bind to MYC

ITGA9 also was a cricial cell adhesion molecule since GSEA results showed that genesets including KEGG ECM receptor interaction, GO cell adhesion via plasma membrane adhesion molecules, NABA ECM glycoproteins, GO calcium dependent cell cell adhesion via plasma membrane cell adhesion molecules, GO protein complex involved in cell adhesion, NABA core matrisome, cell–cell adhesion were enriched in patients with low ITGA9 expression in the GSE2658 (Fig. 7b–h). An enrichment map was constructed using genesets which related to cell adhesion with p‐value < 0.05 (Fig. 7a). Results demonstrated that SCHLOSSER myc targets repressed by serum and hallmark myc targets v1 genesets were enriched in patients with low ITGA9 expression in the GSE2658 (Fig. 7i–j). There was a negative correlation between the mRNA expression of ITGA9 and MYC in multiple cancers based on the GEPIA database (Fig. 7k, p = 7.7e-19, spearman correlation coefficient r = −0.09). We predicted that MYC may bind to the promoter of ITGA9 and repressed its expression based on TF prediction by PROMO (Fig. 7l). In conclusion, ITGA9 was a vital cell adhesion molecule in myeloma which may be negtive regulated by MYC.

### ITGA9 and LAMB1 expression and clinical outcome association in different cancers

We explored the expression and prognosis of ITGA9 and LAMB1 mRNA in different cancers based on the Oncomine database and the PrognoScan database respectively.

**Fig. 8:**
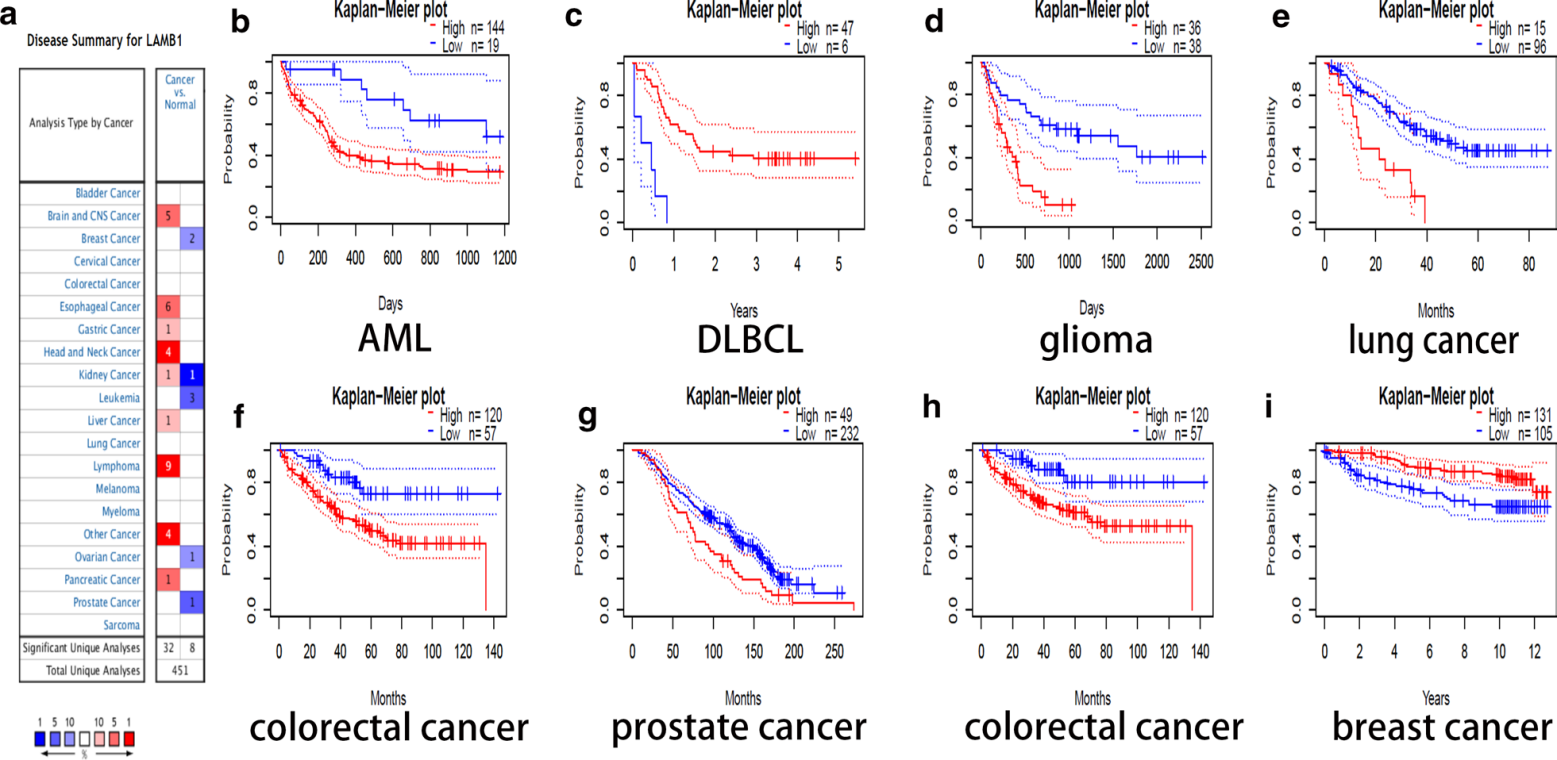
ITGA9 and LAMB1 expression and clinical outcome association in different cancers. **a** LAMB1 expression level in multiple cancers from Oncomine Database. The left box in red indicated the number of datasets with high expression and the right box in blue indicated the number of datasets with low expression after comparing cancers and normal tissues. **b–i** The OS (**b–g**) and DSS (**h–i**) of MM patients with high and low expression of LAMB1 in different cancers was evaluated by **k–m** plots using PrognoScan

LAMB1 is highly expressed in most cancers while low in some cancers including breast cancer, leukemia, ovarian cancer and prostate cancer (Fig. 8a). Furthermore, LAMB1 was significantly associated with OS and DSS respectively in AML, DLCBL, glioma, NSCLC, colorectal cancer (Fig. 8b–g) and colorectal cancer, breast cancer (Fig. 8h–i).

**Fig. 9:**
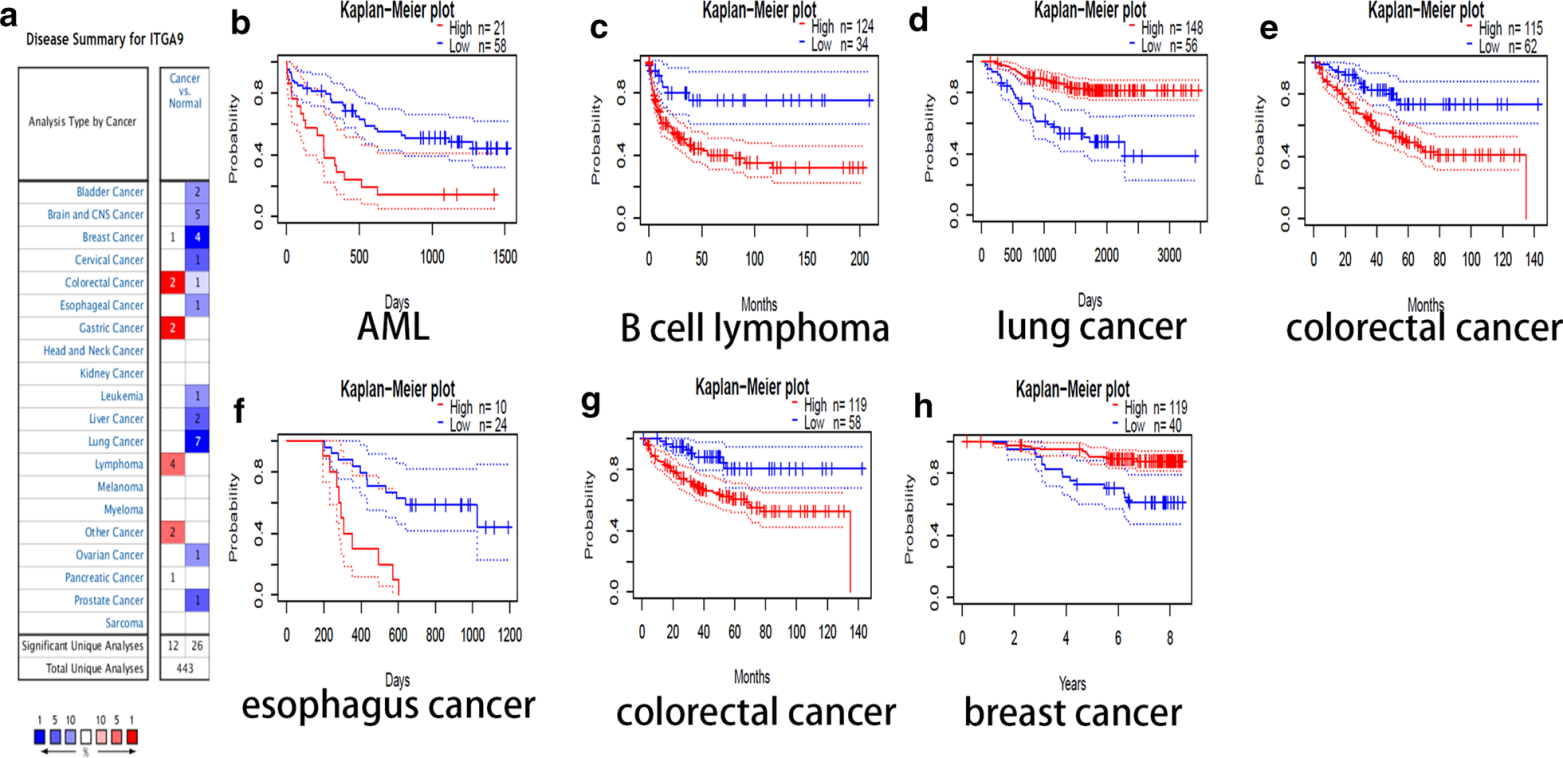
ITGA9 expression in different databases. **a** ITGA9 expression level in cancers in Oncomine Database. (**b–g**) The OS (**b–f**) and DSS (**g–h**) of MM patients with high and low expression of ITGA9 in different cancers was evaluated by **k-m** plots using PrognoScan

Compared with ND, the transcript levels of ITGA9 indicated significant low expression in bladder cancer, brain and central nervous system cancer, breast cancer, leukemia, liver cancer, lung cancer and etc., suggesting that the down-regulation of ITGA9 was common in various types of cancer (Fig. 9a). ITGA9 was significantly associated with OS and DSS respectively in AML, B cell lymphoma, lung cancer, colorectal cancer and esophagus cancer (Fig. 9b–f) and colorectal cancer, breast cancer (Fig. 9g–h). The details including hazard ratio (HR) with 95% confidence intervals(CI) and p-values were shown in the Table 5.

**Table 5:** The details of survival analysis of LAMB1 and ITGA9 in different cancers based on the PrognoScan database

Gene symbol	Cancer type	Subtype	Endpoint	Cohort	Contri-butor	Array type
ITGA9	Breast cancer	-	Disease specific survival	Stockholm (1994–1996)	Pawitan	HG-U133A
ITGA9	Breast cancer	-	Disease specific survival	Stockholm (1994–1996)	Pawitan	HG-U133B
ITGA9	Blood cancer	B-cell lymphoma	Overall survival	Berlin (2003–2005)	Hummel	HG-U133A
ITGA9	Blood cancer	AML	Overall survival	AMLCG (2004)	Metzeler	HG-U133_Plus_2
ITGA9	Colorectal cancer	-	Overall survival	MCC	Smith	HG-U133_Plus_2
ITGA9	Esophagus cancer	Adenocarcinoma	Overall survival	Sutton	Giddings	CRUKDMF_22K_v1.0.0
ITGA9	Lung cancer	Adenocarcinoma	Overall survival	NCCRI	Okayama	HG-U133_Plus_2
ITGA9	Colorectal cancer	-	Disease specific survival	MCC	Smith	HG-U133_Plus_2
LAMB1	Breast cancer	-	Disease specific survival	Uppsala (1987–1989)	Miller	HG-U133A
LAMB1	Colorectal cancer	-	Disease specific survival	MCC	Smith	HG-U133_Plus_2
LAMB1	Blood cancer	DLBCL	Overall survival	GELA (1998–2000)	Jais	HG-U133A
LAMB1	Blood cancer	AML	Overall survival	AMLCG (1999–2003)	Metzeler	HG-U133A
LAMB1	Brain cancer	Glioma	Overall survival	UCLA (1996–2003)	Freije	HG-U133A
LAMB1	Colorectal cancer	-	Overall survival	MCC	Smith	HG-U133_Plus_2
LAMB1	Lung cancer	NSCLC	Overall survival	Duke	Bild	HG-U133_Plus_2
LAMB1	Prostate cancer	-	Overall survival	Sweden (1977–1999)	Sboner	6K DASL

Probe ID	Number	COX P-Value	ln (HR)	HR [95% CI]	Dataset
206009_at	159	0.028871	−0.63283	0.53 [0.30–0.94]	GSE1456-GPL96
227297_at	159	0.003648	−0.73661	0.48 [0.29–0.79]	GSE1456-GPL97
206009_at	158	0.01101	1.61013	5.00 [1.45–17.31]	GSE4475
227297_at	79	0.000647	0.362666	1.44 [1.17–1.77]	GSE12417-GPL570
1555335_at	177	0.042238	−0.7844	0.46 [0.21–0.97]	GSE17536
1692944	34	0.008685	2.15512	8.63 [1.73–43.15]	GSE11595
227297_at	204	0.012868	−0.64768	0.52 [0.31–0.87]	GSE31210
1555335_at	177	0.03298	−0.93029	0.39 [0.17–0.93]	GSE17536
211651_s_at	236	0.048855	−0.67082	0.51 [0.26–1.00]	GSE3494-GPL96
201505_at	177	0.00468	0.735706	2.09 [1.25–3.47]	GSE17536
201505_at	53	0.032997	−0.47212	0.62 [0.40–0.96]	E-TABM-346
211651_s_at	163	0.042439	1.21397	3.37 [1.04–10.88]	GSE12417-GPL96
201505_at	74	0.001456	0.49603	1.64 [1.21–2.23]	GSE4412-GPL96
201505_at	177	0.008075	0.587218	1.80 [1.17–2.78]	GSE17536
211651_s_at	111	0.003862	0.844036	2.33 [1.31–4.12]	GSE3141
DAP2_5968	281	0.0261	0.293184	1.34 [1.04– 1.74]	GSE16560

## Discussion

MM is a common hematological malignancy so it is vital to investigate the molecular mechanisms. Microarray has been widely used to analyze the expression changes of genes in MM and predict the potential biomarkers.

In this study, we analyzed gene mRNA expression data from BM plasma cells in GSE6477 dataset using GEO2R and revealed that there were 1383 DEGs between MM patients and NDs, consisting of 538 upregulated genes and 845 downregulated genes with p < 0.05 and [logFC] ≥ 1. Furthermore, we performed GO and KEGG enrichment analyses to explore main function of DEGs. GO enrichment analysis in BPs recognized the functional enrichment of DEGs in the immune response, inflammatory response, ECM organization, leukocyte migration, and cell adhesion. KEGG analysis showed enrichment of hematopoietic cell lineage, complement and coagulation cascades, CAMs, proteoglycans in cancer, ECM-receptor interaction and PI3K/Akt signaling pathway. It was quite clear that the loss of cell–cell adhesion was an important event for acquiring the invasive and metastatic phenotype. These results indicated the importance of cell adhesion in MM progression.

We screened overlapped genes between DEGs and genes in GO terms of cell adhesion and constructed a PPI network. The 12 hub genes were listed: ITGB1, FN1, ITGB3, ITGAM, PTPRC, ITGB2, ITGA5, ITGB5, CDH1, IL4, ITGA9, and LAMB1. Among the 12 hub genes, ITGAM, ITGB2, ITGA5, ITGB5, CDH1, IL4, ITGA9 and LAMB1 had diagnostic value, which can distinguish MM from normal people. The mRNA expression of ITGA9 and LAMB1 were significantly associated with the OS and DSS of MM patients. These results suggest that ITGA9 and LAMB1 exhibit both diagnostic and prognostic values in MM. Further investigations regarding to the role of LAMB1 and ITGA9 were required.

Here, we found that LAMB1 was abnormally expressed and was associated with OS and DSS in many cancers, which were consistent with previous results. LAMB1 has a high protein level in high-grade gliomas, suggesting a possible correlation with tumor progression [24]. What’s more, LAMB1 was identified to take part in cell attachment and have the capacity to inhibit metastasis. In prostate cancer, LAMB1 was shown to be involved in cell motility and invasion into the surrounding ECM [19]. In our study, low LAMB1 expression was significantly associated with high β2-MG concentration and high R-ISS stage in MM. GSEA results showed that LAMB1 was involoved in cell adhesion and may be induced by hypoxia. TF prediction and the correlation analysis suggested that HIF-1 may bind to LAMB1 promoter to increase its transcription.

ITGA9 abnormal expression was found in many cancers and was likely to correlate with higher grade cancers [9]. For example, sequencing analysis also found that ITGA9 was significantly down-regulated in cervical squamous cell carcinoma [22]. Genetic variation and epigenetic modification of ITGA9 are related with the tumorgenicity and progression of colorectal cancer [23]. In our study, we found down-regulated ITGA9 was associated with poor outcome in MM. Myeloma patients with low ITGA9 expression had more tendency of having the higher number of malignant PCs in BM and becoming the higher GEP group. GSEA results showed that ITGA9 was a vital cell adhesion molecule and may be repressed by MYC in myeloma. Based on the TF prediction and the correlation analysis, we predicted MYC may bind to the promoter of ITGA9 for transcriptional repression.

## Conclusions

We identified eight hub genes, including ITGAM, ITGB2, ITGA5, ITGB5, CDH1, IL4, ITGA9, and LAMB1 to be potential diagnostic markers in MM. Further study demonstrated ITGA9 and LAMB1 which correlated with clinical characters and prognosis may play important roles in the cell adhesion and can be regulated by different TFs in MM.

## Data Availability

The datasets used and/or analyzed during the current study are available from the corresponding author upon reasonable request.

## Abbreviations

AUC: Area under the curve
BM: Bone marrow
CAMs: Cell adhesion molecules
CDH1: Cadherin 1
DAVID: Database for annotation, visualization and integrated discovery
DEG: Differentially expressed gene
DSS: Disease-specific survival
ECM: Extracellular matrix
FDR: False discovery rate
FN1: Fibronectin 1
GEO: Gene expression omnibus
GEP: Gene expression profile
GEPIA: Gene expression profiling interactive analysis
GO: Gene ontology
GSEA: Gene enrichment analysis
IL4: Interleukin 4
ITGA: Integrin subunit alpha
ITGB: Integrin subunit beta
KEGG: Kyoto encyclopedia of genes and genomes
K-M: Kaplan-meier
LAMB1: Laminin subunit beta 1
MCODE: Molecular complex detection
MGUS: Monoclonal gammopathy of undetermined significance
MM: Multiple myeloma
ND: Normal donor
NES: Normalized enrichment score
PI3K: Phosphatidylinositide 3-kinases
PPI: Protein-protein interaction
PTPRC: Protein tyrosine phosphatase receptor type c
R-ISS: Revised-international staging system
RMM: Relapsed mm
ROC: Receiver operatoring characteristic
SMM: Smoldering multiple myeloma
STRING: Search tool for the retrieval of interacting gene
TF: Transcription factor
